# Supply-related drivers of staff motivation for providing intermittent preventive treatment of malaria during pregnancy in Tanzania: evidence from two rural districts

**DOI:** 10.1186/1475-2875-11-48

**Published:** 2012-02-18

**Authors:** Godfrey M Mubyazi, Paul Bloch, Jens Byskov, Pascal Magnussen, Ib C Bygbjerg, Kristian S Hansen

**Affiliations:** 1 National Institute for Medical Research (NIMR), Centre for Enhancement of Effective Malaria Interventions (CEEMI), 2338 Ocean Road, P.O Box 9653, Dar-es-Salaam, Tanzania; 2 Amani Medical Research Centre, P.O Box 81 Muheza, Tanzania; 3 DBL-Centre for Health Research and Development, Faculty of Life Sciences, University of Copenhagen, Bulöwsvej, 1870 Fredriksberg, Copenhagen, Denmark; 4 Steno Health Promotion Centre, Steno Diabetes Centre, Gentofte, Denmark; 5 Institute of International Health, Immunology and Microbiology, Faculty of Health Sciences, University of Copenhagen, Bleddamsvej 3, DK 2200 Copenhagen N, Denmark; 6 London School of Hygiene and Tropical Medicine, Keppel Street WC1, London, UK

**Keywords:** Human resources, Health worker motivation, Malaria, Health-care services, Malaria in pregnancy, Tanzania

## Abstract

**Background:**

Since its introduction in the national antenatal care (ANC) system in Tanzania in 2001, little evidence is documented regarding the motivation and performance of health workers (HWs) in the provision of intermittent preventive treatment of malaria during pregnancy (IPTp) services in the national ANC clinics and the implications such motivation and performance might have had on HWs and services' compliance with the recommended IPTp delivery guidelines. This paper describes the supply-related drivers of motivation and performance of HWs in administering IPTp doses among other ANC services delivered in public and private health facilities (HFs) in Tanzania, using a case study of Mkuranga and Mufindi districts.

**Methods:**

Interviews were conducted with 78 HWs participating in the delivery of ANC services in private and public HFs and were supplemented by personal communications with the members of the district council health management team. The research instrument used in the data collection process contained a mixture of closed and open-ended questions. Some of the open-ended questions had to be coded in the form that allowed their analysis quantitatively.

**Results:**

In both districts, respondents acknowledged IPTp as an essential intervention, but expressed dissatisfaction with their working environments constraining their performance, including health facility (HF) unit understaffing; unsystematic and unfriendly supervision by CHMT members; limited opportunities for HW career development; and poor (HF) infrastructure and staff houses. Data also suggest that poor working conditions negatively affect health workers' motivation to perform for ANC (including IPTp) services. Similarities and differences were noted in terms of motivational factors for ANC service delivery between the HWs employed in private HFs and those in public HFs: those in private facilities were more comfortable with staff residential houses, HF buildings, equipment, availability of water, electricity and cups for clients to use while taking doses under direct observed therapy than their public facility counterparts. Employees in public HFs more acknowledged availability of clinical officers, nurses and midwives than their private facility counterparts. More results are presented and discussed.

**Conclusion:**

The study shows conditions related to staffing levels, health infrastructure and essential supplies being among the key determinants or drivers of frontline HWs' motivation to deliver ANC services in both private and public HFs. Efforts of the government to meet the maternal health related Millennium Development Goals and targets for specific interventions need to address challenges related to HWs' motivation to perform their duties at their work-places.

## Background

Maternal deaths are still high in Tanzania. Records show that the maternal mortality rate is 454 per 100,000 live births while the infant mortality rate is estimated at 51 per 1,000 live births, a significant proportion of these deaths being related to malaria [1]. The provision and coverage of preventive and treatment services, including those addressing maternal and child health (MCH), including antenatal care (ANC), are constrained by fragile health systems [2]. Malaria chemoprevention during pregnancy, commonly known as intermittent preventive/presumptive treatment of malaria during pregnancy (IPTp), was introduced in the Tanzanian health system under ANC services in 2001. The guideline on IPTp requires administration of at least two doses of sulphadoxine-pyrimethamine (SP) during the second and third trimester of pregnancy [3,4]. In 2000 the African Heads of State through their meeting in Abuja made a Declaration by setting a target of making every effort possible to ensure that 60% of the pregnant women attending ANC clinics in their countries were covered by IPTp by receiving two doses of SP during their pregnancy [5]. However, new targets had been set by the Ministry of Health and Social Welfare (MoHSW) of Tanzania with reference from targets reset by the Roll Back Malaria (RBM) program supported by the World Health Organization (WHO) and other partners. For instance, Tanzania has set a target of attaining 80% coverage of pregnant women attending ANC clinics with IPTp-SP by 2010-2013 (Dr. MufungoMarero, Head of Case Management of Malaria and Malaria In Pregnancy Unit, NMCP, Tanzania, per comm.).

Few studies have evaluated the ability of the healthcare system to embrace IPTp. As documented by other authors, reviews of evidence from systematic studies indicate contextual demand and supply related constraints being a challenge to effective implementation of the IPTp strategy among other maternal and child health (MCH) and ANC services [6-8]. Little is known on demand and supply side factors directly or indirectly influencing the IPTp service delivery and coverage [7], and this includes the factors affecting the motivation and performance of the frontline health workers (HWs).

The motivation and commitment of HWs affect the quality of services and the satisfaction of clients, and are thus important for successful implementation of MCH programmes and health policies of a nation [9,10]. Much has been discussed about the quality of MCH services across sub-Saharan Africa (SSA) [7,11]. Where services are of poor quality a relationship is found to supply factors, such as HW behaviour towards clients, drug shortages, poor remuneration, poor health facility (HF) infrastructure, and low HW knowledge and skills [3,11]. Reports from Zambia, Uganda and Tanzania show that HF understaffing has intensified the workload of staff and affecting quality of care [8]. Experts have warned that serious shortage of skilled and motivated HsW is an important challenge toward attainment of the Millennium Development Goals for health [12].

The need for research on the feasibility of interventions aimed at reducing the burden of malaria during pregnancy including IPTp under prevailing resource constraints in most malaria endemic countries, especially those in SSA, is generally acknowledged [13]. This includes paying attention to the experience, perceptions and concerns of HWs responsible for implementing newly recommended interventions [14], often challenged by inadequate supplies, logistics, manpower numbers, skills and morale [15-18]. For the purpose of contributing to the knowledge base on health sector performance, the present paper describes supply related factors that may influence the motivation of HWs engaged in the provision of ANC services in general and specifically in IPTp services in Tanzania using a case study of two districts that are predominantly rural and located in two different regions.

## Methods

### Study design

This paper is part of dissemination of the information from a large cross-sectional case study carried out to find out the economic and other contextual determinants of the acceptability and practicability of IPTp services in Tanzania using a case study of two districts namely, Mkuranga and Mufindi and was carried out between January 2005 and February 2007. However, data for the currently reported sub- study were collected between November 2005 and October 2006 with additional personal communications with national level managers made between September 2011 and January 2012.

In the larger study, different data collection and analytical techniques targeting different categories of informants, the aim being to obtain data from multiple sources of evidence were applied, and among the individuals approached were the HWs involved in the delivery of ANC [3]. The location, characteristics and criteria used for selecting the study districts have been described elsewhere [19].

### Sampling approaches

In total, 78 HWs involved in the delivery of ANC services were selected from the two study districts, particularly 43 from Mufindi and 35 from Mkuranga. The HWs were identified at private-for-profit (commercial) HFs, faith-based HFs (private-not-for-profit, sometimes known as 'mission facilities') and government/public HFs using a mixture of probability and non-probability sampling techniques. Either a simple random sampling method or convenient sampling method was applied in the selection of HFs and HWs for study, and this depended on the number of HFs and HWs who were present/available to participate during the study implementation days/period. Thus, where the targeted HF was found with less than four HWs involved in ANC work as in most rural dispensaries, a convenient sampling method was applied to select at least two of them. A targeted HF found with four or more HWs participating in delivering ANC (including IPTp) services allowed the application of random sampling method for selecting more than two HWs for the study. In total 28 HFs were identified in both districts and such facilities involved commercial, faith-based and public facilities. The selected HFs included hospitals, health centres and dispensaries. The number and types of such facilities and their locations in the study districts and from two regions as well as their selection criteria have already been explained elsewhere. But in brief, the selected study districts are located in different regions, each in the zonal area with different malaria transmission intensities, socio-economic characteristics and health infrastructure profiles, although both districts were typically rural [19]. Mkuranga district capital is located at just 60 km from the city of Dar Es Salaam where the headquarters of the MoHSW is located while Mufindi is at about 483 km from Dar Es Salaam.

Included in the study were clinical officers (one from each HF studied), nursing officers, midwives (NOs), laboratory personnel, nurse auxiliaries (officially known as medical attendants in Tanzania), public health nurses (PHNs), maternal and child health aides (MCHA), and health assistants. In Mkuranga, 20 HWs were identified from public HFs while 15 workers were identified in private facilities. In Mufindi, 30 HWs were identified in public facilities while 13 were identified in private facilities. The majority 51 (65.4%) of the workers were found in the dispensaries, 16 workers came from health centres, and 11 came from hospitals. Also, most (79.5%) of the workers were females. The mean number of years the individual respondents had stayed at particular study HF was 8.3, the range of period of staying being 0.5-24 years.

In terms of hierarchy, COs are highly ranked because of nature (or by virtue) of their training backgrounds as they work to assist in doing clinical diagnoses, patients prescriptions and delivering other services of medical/clinical nature which are not performed by (or are not a duty of) the rest of the staff cadres. Midwives and nurses are sometimes difficult to distinguish due to the nature of their training as they almost go through the same orientations at schools/colleges, the difference comes when one of the two groups concentrates/specializes in the delivery of services to specific categories of patients/clients at HF levels. Specifically, midwives mainly work in maternity wards and MCH clinics dealing with mothers and have nursing training backgrounds also. Therefore, they have specialty of care for women and working under guidance by doctors or clinic officers to help guide the patients through their pregnancy and birth process, are involved in activities such as counseling and educating patients, conducting physical examination, delivering babies and attending other pregnancy related issues including performing some diagnostic tests, but for Tanzania unlike in some other countries, they are not responsible for prescribing pharmacological treatment. The rest of nurses (i.e. NOs) perform general nursing duties as performed by the midwives, particularly of general obstetric nature, but as are some of the medical attendants, they do work in sections such as paediatric wards, surgical sections/wards, and other patients wards of general or specific service nature. However, the nature of work done by these groups sometimes depends on the conditions confronted at HF levels, for instance, the number of skilled workers available to deliver the desired services, otherwise it is not uncommon to find a midwife working in the ANC clinic or other sections at HF level like the PHNs, MCHA and some medical attendants (Dr. MufingoMarero, NMCP and Dr. Judith Msovela, NIMR-Center for Enhancement of Effective Malaria Interventions, per communication).

### Data collection

Data collection included i) field observations of HF conditions such as office space, state of repair of the existing old buildings, waiting rooms for the patients/clients, availability of water, electricity and condition of toilets, number of HWs present at particular HF level during the time of study visit to the HF, among other things. This was made possible through the use of a structured observation checklist by the well oriented data collectors based on the experience gained during the pre-testing of the research instruments and pilot phases of the study that were carried out in peripheral areas of two districts in Dar Es Salaam, namely Ilala and Temeke as well as some parts of Mkuranga district. The data collectors were oriented to observe what were in place and judge by themselves and where necessary/possible ask the existing personnel on their views regarding the implications of the condition observed on the motivation of the frontline HWs to deliver the required services;(ii) review of HF documents including supervisory visits paid by the members of the district council health management team (CHMT), inventory records, for instance, drugs (including the SP and antipyretics) seeming to be out of stock; (iii) personal communications with members of the CHMT. The communication involved questions aimed at seeking clarifications or opinions about several requests or concerns raised by HWs in relation to their day-to-day IPTp related activities, for instance, whether the conditions of the HF infrastructure, training qualifications of HWs involved in service delivery and supplies had any influence on HWs' motivation and performance in relation to IPTp administration. However, the CHMT also responded to the separate in-depth interview focusing on more broad national policy, planning and health systems related issues of malaria preventive interventions that have been presented in a separate paper submitted elsewhere for peer review and possible publication. iv) a questionnaire with a mixture of closed- and open-ended questions was administered by the interviewers to the HWs. The closed questions were aimed at collecting direct quantitative data while the open-ended questions were aimed at obtaining explanations or clarifications on particular issues of interest so as to provide information that corroborated the answers obtained from the closed questions. The interviews for the currently reported sub-study addressed the issues related to the respondents' experiences, perceptions and attitudes towards delivery of ANC services including administration of IPTp doses to the ANC clients under the existing working conditions. This included financial and non-financial factors considered to possibly have had a bearing (influence) on HWs' motivation for delivering ANC (including IPTp) services. For instance, HWs were asked about the degree to which they were satisfied with conditions of health facility infrastructure, residential houses for the HWs, staff promotion procedures and their translation into practice, quality of HW supervision carried out by the CHMT members, HW remuneration, and availability of necessary supplies such as drugs including SP for IPTp delivery free-of-charge to users, laboratory conditions, and documents containing ANC service delivery guidelines. To arrive at inclusion of these factors or variables in the assessment, reference was made to studies and analyses published elsewhere, for instance, Lapane and Hughes [15], Dielman *et al. *[16,17], Dussault and Dubois [18], to mention some. Included in the research instrument also was the nature of the services provided, for instance, whether health talks and education related to IPTp services and advice on safe motherhood and proper drug use during pregnancy were being provided to the pregnant women attending ANC clinics.

### Data processing and analysis

Qualitative data based on answers obtained from the respondents to open-ended questions were analyzed manually-the principal author in this paper who was a PhD student by that time had adequate time for accomplishing this because after all the data collected were not too large to be analyzed manually despite having also had consulted other social scientist with skills and experience using this approach. The approach was to identify the meaningful content(s) of the information collected, organized into codes and interpreted in triangulation along with results from quantitative data. The latter type of the data was processed with the aid of STATA 8.2 (Texas Inc., USA) computer software program to obtain descriptive results that were displayed in form of one-way frequency distribution tables, cross-tables based on Spearman's Chi-square tests for relatively larger sample responses or Fisher's exact test for small samples indicating less than thirty responses or number of observations. Inference that statistically significant association between the identified dependent and independent (predictor) variables existed was arrived made if a p-value resulting from a statistical test made was less than or equal 0.05 at 95% confidence interval (CI). The variables that were included in the statistical tests of associations between dependent variables and explanatory variables include those that seemed relevant to have possible influence on HWs' motivation for delivery of the desired services based on the analysts' experience and view. This is scientifically acceptable [20], even though in the first place questions may be raised by critics regarding the inclusion of some of the variables in the statistical tests or logistic regression models. For instance, an unmotivated HW may not value the need for providing some important services such as health education or health talks to the clients especially when the type of the health service concerned seems to be associated with several delivery procedures that are quite involving the HW. Thus, since motivation is multi-dimensional and involves a range of financial and non-financial incentives [21], a proxy variable was chosen as an indicator of motivation and this proxy emphasized on HWs' perceptions based on the questions posed to them to express their positions in terms of the extent to which they were feeling "comfortable/satisfied with the working conditions" at their work-places. The degree to which the satisfaction influenced the performance of such workers in relation to the delivery of ANC (including IPTp) services was generally addressed in qualitative terms. Data interpretation was made in consideration of possible design effects that might have emanated from the different samples of HF levels owned by different authorities (private, FBO, government) and the type of technique used in the data analysis.

## Results

### Health workers' general motivation

Although as explained above several questions were posed in attempt to assess individual HWs' motivation for delivering ANC services in general and specifically for IPTp, only 3(8.6%) and 8(18.6%) of the respondents in Mkuranga and Mufindi, respectively, expressed to be completely (or fully) comfortable with all the working conditions surrounding the facilities in which they were found working; the rest were either not comfortable at all, or a little comfortable and had expressed several reservations related to the following elements:

• Understaffing at their work-places which increase workload to the existing staff who at times due to exhaustion/tiredness fail to deliver some of the services as required

• Unsystematic and unfriendly supervision (less supportive supervision) by district health management teams, especially to the peripheral HWs who were less disadvantaged in their working environment than their urban-based counterparts

• Limited opportunities for staff career development especially for those working in the private sector. This includes the chances for training on focused ANC (fANC) and other MCH services issues

• Poor/limited HF infrastructure such as working space, conditions of toilets in terms of safety due to poor/lack of roofing and doors, shortage of water supply for washing after self-relieving and water for washing equipment and drinking at HF level both for patients and HWs, and lack of (or un-rehabilitated) staff houses

### Understaffing at health facilities

All of the respondents from both districts reported understaffing as the most critical discouraging factor and a hindrance to effective practice of DOT in relation to IPTp administration and in for the provision of other MCH services in current words, the reproductive and child (RCH services). Respondents, especially from Mkuranga admitted failure of the remaining few HWs who are left at HF levels to carry out all the required fANC and other RCH services on particular days when their fellow HWs travel to town for collecting their monthly salaries or healthcare service materials from the DMO's Office or when they move away for mobile/outreach clinic services in the district. At one time recently, the government announced its plan to phase out the MCH Aides (MCHA) and Medical Attendant cadres who have not upgraded their skills with the view to ensuring that HFs were staffed with more qualified/trained personnel. This strategy was underscored by the respondents on ground that it would negatively affect the volume and quality of MCH services in general because already most HFs are understaffed in terms of the midwives and other skilled nurses while the few available COs have not always been performing such MCH service duties as the administration of SP to pregnant women for IPTp under DOT, vaccination and other RCH services, and their main duty was to deal with clinical aspects including patient diagnoses and treatment procedures. Thus, respondents from both districts warned that phasing out the Medical Attendant and MCHA cadres would aggravate career development opportunities and motivation for these cadres in particular in relation to MCH services and the human resources crisis in the health sector in general. It was lamented from both districts that already such staff cadres working in the public sector were demoralized to work to their full capacity after hearing such news given the fact that some were already too old to go to colleges to attend further career development courses while the rest majority had little prospects for acquiring opportunities for upgrading their skills.

Looking at the results from bivariate analyzes (Table 1) it was observed that at 95% CI, the HWs who were found in government facilities were: (i) about 4 times more likely than their counterparts at private HFs to express being satisfied with the availability of COs, the observed difference was statistically significant (OR = 3.68; *p *= 0.035; CI: 1.10-12.24); (ii) less likely to be satisfied with presence of lab personnel, also the difference being statistically significant (OR = 0.05; *p *= 0.006; CI: 0.01-0.43); (iii) a little times more likely to be satisfied with presence of midwives (OR = 1.83; *p *= 0.397; CI: 0.45-7.40) and public health nurses (PHNs) (OR = 1.13; *p *= 0.925; CI: 0.09-12.99), but as shown in both of these cases the observed differences were not statistically significant. Also considering general working conditions (including all elements of the services required such as staff numbers and skills, infrastructure, essential supplies, remuneration and other no-financial factors), the respondents who were found in government HF were more likely to express being satisfied with the available number of MCH Aides than their counterparts in private facilities, although the difference in the two latter cases were not statistically significant (OR = 0.54; *p *= 0.551; CI: 0.07-4.07). However, the multivariate regression analysis involving a set of predictor variables (e.g. availability of key health workers, essential supplies such as drugs for IPTp, opportunities for attending any training program as part of career development or in-service knowledge upgrading; availability of staff residential houses and other health infrastructure conditions) did not identify any of such variables to be a statistically significant driver of the HWs' motivation to deliver the ANC services or as a factor seeming to have a significant association with the HWs' expression of being satisfied with the listed working conditions. Additional findings regarding the respondents' expressions about the satisfaction with the working conditions are also presented (Table 2).

**Table 1:** Bivariate regression analysis of statistical association between the place of work of health workers (the dependent variable)) and their satisfaction with staffing conditions (the independent variables).

Independent variables	Frequency	*P*-value	OR	95% CI
1. Satisfied with the number of NOs	12 (15.4%)	0.397	1.83	0.45-7.40

2. Satisfied with the number of public health nurses	3 (3.8%)	0.925	1.13	0.09-12.99

3. Satisfied with the number of COs	23 (29.5%)	0.034	3.68	1.10-12.24

4. Satisfied with the number of laboratory staff	9 (11.5%)	0.006	0.05	0.01-0.43

5. Satisfied with the number of MCHA	4 (5.1%)	0.551	0.54	0.07-4.07

The number and percentage of HW confirming their satisfaction is presented in the "Frequency" column (N = 78). The odds ratios (OR) and 95% confidence intervals (CI) are calculated for health workers employed at a public/government HF at the time of the interview. HWsfound at a private HF serve as the reference group. Data are combined for the two target districts

**Table 2:** Bivariate regression analysis on statistical associations between the perceived motivation of frontline HWs and nine independent variables.

Dependent variable: "Do you personally feel	Frequency	OR	*P*-value	95% CI
comfortable or satisfied with the working				
conditions in your current work?"				
1. Working at a government HF	50 (64.1%)	1.59	0.523	0.39-6.54

2. Being a CO	21 (26.9%)	1.02	0.977	0.24-4.28

3. Being a trained or qualified nurse (i.e. nurse midwive, PHN or NO)	19 (24.4%)	.65	0.609	0.13-3.33

4. Being a medical attendant or a MCHA	36 (46.2%)	1.48	0.549	0.41-5.33

5. Having received training in fANC	47 (60.3%)	.76	0.677	0.21-2.75

6. Having received specific training on IPTp services	36 (46.2%)	.68	0.549	0.19-2.43

7. Knowing the criteria used in the selection of staff for IPTp training	31 (39.7%)	2.02	0.285	0.56-7.29

8. Having been promoted within the last three years	20 (25.6%)	2.89	0.115	0.77-10.79

9. Knowing the criteria used in the promotion of HW	42 (53.9%)	2.59	0.186	0.63-10.61

"Frequency" indicates the number of "yes" answers to the independent variables. Data are combined for both districts (N = 78). OR is the odds ratio while CI is the confidence interval. The p-values are indicated just for easy noting of the statistical significance of the differences tested/shown (cf. Chapter 4)

Counter-intuitive results were obtained from the assessment of the degree to which the individual respondents expressed to be satisfied with general working conditions based on their response to particular study questions. It was observed that: (i) the MCHA, lab attendants, medical attendants and health assistants-generally these four groups considered to be semi-skilled/qualified workers were about 1.5 times more likely to be satisfied than the skilled (i.e. the trained staff including COs, midwives, NOs, and PHNs (OR = 1.48; *p *= 0.549; CI: 0.41-5.33). That is, from the analysts' viewpoint, trained staff cadre was expected to be more satisfied due to their being in position to receive better remuneration and obtaining more opportunities related to their work (e.g. attending seminars and other official trips) by virtue of their qualifications than the less skilled staff cadre; (ii) those who were informed about the criteria for appointing the HWs to attend seminars/workshops (or short courses) related to focused ANC (including IPTp) services were about 2 times more likely to be satisfied than those uninformed (OR = 2.02; *p *= 0.285; CI: 0.56-7.29); (iii) those who had been promoted in the last three years prior to this study were about 3 times more likely to express satisfaction than those not promoted (OR = 2.89; *p *= 0.115; CI: 0.77-10.79); (iv) those who were informed about the criteria used for promoting staff were about 3 times more likely to be satisfied than those uninformed (OR = 2.59; *p *= 0.186; CI: 0.63-10.61). Additional data are presented, including satisfaction with opportunities for training in relation to fANC (in which IPTp is part of the) services (Table 3).

**Table 3:** Bivariate regression analysis of statistical association between the place of work of HWs (dependent variable) and their satisfaction with various working conditions (the predictor variables).

*Independent variables*	*Frequency*	*P-value*	*OR*	*95% CI*
1. Satisfied with the availability and condition of residential houses for HWs	14 (17.9%)	0.001	0.09	0.02-0.39

2. Satisfied with the physical condition of the buildings of the HFas a whole	31 (39.7%)	0.368	0.65	0.25-1.66

3. Satisfied with the availability and condition of furniture at the HF as a whole	23 (29.5%)	0.004	0.22	0.08-0.62

4. Satisfied with the availability and condition of furniture specifically at the ANC clinic	27 (34.6%)	0.001	0.16	0.06-0.45

5. Satisfied with the number and condition of consultation rooms at the HF as a whole	9 (11.5%)	0.865	1.14	0.26-4.94

6. Satisfied with the number and condition of toilets for use by staff and clients around the HF	40 (51.3%)	0.088	0.44	0.02-1.13


7. Satisfied with the physical condition of the laboratory at the HF	17 (21.8%)	0.030	0.29	0.09-0.89

8. Satisfied with the availability of essential laboratory supplies at the HF	30 (38.5%)	0.120	0.47	0.18-1.22

9. Satisfied with the availability and condition of drug storage facilities at the HF	42 (53.9%)	0.169	0.51	0.19-1.33

10. Satisfied with the availability of water at the HF	31 (39.7%)	0.021	0.32	0.12-0.84

11. Satisfied with the availability and reliability of electricity at the HF	24 (30.8%)	0.028	0.32	0.12-0.88

12. Satisfied with the availability of cups for practicing DOT at the ANC clinic	29 (37.2%)	< 0.001	0.09	0.03-0.26

13. Satisfied with the availability of SP for IPTp services at the HF/ANC clinic	49 (62.8%)	0.100	0.42	0.15-1.18

14. Satisfied with the availability of antipyretics at the HF/ANC clinic	36 (46.2%)	0.056	0.39	0.15-1.02

The number and percentage of HWs confirming their satisfaction is presented in the "Frequency" column (N = 78). The odds ratios (OR) and 95% confidence intervals (CI) are calculated for the HWs employed at a public/government HF at the time of the interview. HWs at a private HF serve as the reference group. Data are combined for the two target districts

### Provision of health education

Despite the understaffing situation facing most of the health facilities, 27 (77.1%) and 36 (83.7%) of the respondents in Mkuranga and Mufindi districts, respectively, confirmed to have been giving messages related to IPTp with SP regularly/always to their clients as a routine practice. Informing the clients about the possible side effects of SP was sometimes not done as an attempt to avoid scaring their clients who already might have had perceived SP negatively and who might avoid taking SP in the future, as testified by seven (25.9%) and three (8.3%) of the respondents in the latter two districts, respectively. This issue was investigated in attempt to assess the compliance of the respondents with the recommended guidelines bearing in mind that a HW who is demoralized at workplace even for something of a general nature within his/her working environment may not value the need for providing useful health education and advisory services through counseling as recommended to the service clients attended at the HF.

### Supervision

Regular provision of supportive supervision of the frontline HWs including aspects related to delivery of ANC services (among which is the IPTp administration issue) was mentioned to be a strong motivational factor since through supervision HWs would get clarification and technical demonstration on issues around health service delivery. As reported by the respondents in both districts and confirmed through review of the government health documents under the health management information system, popular as Mfumo wa Taarifa za Undeshaji wa Husuma za Afya (MTUHA) in Swahili, each HF must be visited for supervision at least once each quarter of the year (every three months) by members of district CHMT, but this does not happen regularly to peripheral HFs which are difficult to reach because of poor roads in both districts and lack of reliable boats for crossing the Indian Ocean to reach some islands in Mkuranga district.

Although almost all [34 (97.1%)] of the respondents in Mkuranga and 43 (100%) of their counterparts in Mkuranga admitted to have been supervised at their work-places by the district CHMT members, 11(31.4%) and 13(30.2%) of the respondents in these districts, respectively, stated to be dissatisfied with (i) the frequency of supervisory visits paid to their health facilities; (ii) surprise supervision seeming to be outside the planned supervision schedule, and (iii) quality of the supervision, including the manner the supervisors behaved to their subjects and failure by some supervisors to give feedback on the previous supervisory report/visits. Preference by most CHMT members to supervise particular HFs more regularly than others was pinpointed a discouragement to HWs who feel ignored.

As reported mostly by the HWs living in remote settings, there has been a deliberate move by the supervisors to avoid visiting the HFs where they anticipate or are informed about existence of problems that were more severe, chronic and less manageable. In contrast, respondents who were more centrally working, particularly those working in urban stations/centres within or near the district headquarters perceived remote facilities as being more favoured in terms of routine supervision. As argued, in many cases supervisors take notes about the expressed concerns but do nothing afterwards and are often too judgmental by looking at mistakes instead of giving the necessary support of guidance. One of the worst things noted as an example is the supervisors who tend to pick the MTUHA registers from the lower level HFs on ground of inspecting them or taking some data for reporting or planning purposes at district or national level but either take a long time before returning them or misplace them by not returning them completely. As a result, people who come later needing to inspect such data either for health service management use at HF level or for evaluation (including research or consultancy) purpose end up missing the required records.

The respondents from the faith-based facilities in both study districts felt largely ignored and complained against what they called district CHMT members (all of which being government employees) showing negative attitudes (or little cooperation) towards private health service providers. Such CHMT members have been condemned for expressing mistrust in the ability of the private sector employees to perform their health duties in conformity to the stipulated national policy guidelines. Nevertheless, a number of them acknowledged some of the supervisors from CHMT especially the district medical officers (DMOs) and district Reproductive and Child Health coordinators (DRCHCos) who seemed to do their jobs well as expected by their sub-ordinate staff at health facility levels. For instance, those who gave notice about their planned visit before they actually paid a visit to the respective HFs or those who pass by the HFs to say hullo and discuss with or help the frontline workers on various logistical issues outside the planned day for supervision while being on their way from/to visiting other facilities were greatly commended to motivate the supervised/visited personnel.: *The District Medical Officer *(DMO) *may pass-by to say hello and listen to other problems even when he is on the way for activities other than supervision.... all the staff like him... since majority of other supervisors are always in haste, rushing*" [Respondents in Mufindi. Similar qualities as mentioned for the DMO was also reported about the DRCH-Co and DMO of Mkuranga].

### Career development and training opportunities

Career development including short-term on-job (in-service) training was viewed as being closely linked with motivation of HWs as it gives the staff added qualifications that are used as basis for their promotion at work and increments in their remunerations, leave alone equipping the staff with the skills required for service delivery as well as having positive effects on the overall performance of the frontline HWs in the delivery of various services including those related to IPTp administration.

HWs' disappointment with not having been promoted in the last three years while they were involved in routine ANC service delivery was expressed by 23(65.7%) and 34(79.1%) of the respondents in Mkuranga and Mufindi districts, respectively. A more disappointment was expressed by the HWs belonging to the junior staff cadres particularly the semi-skilled HWs who on their side reported to have had very fewer opportunities for being invited/nominated to undergo in-service training than the COs and nurses. Also, discouragement with staff promotion was much more expressed by the more matured workers who saw the younger staff being promoted based on the criterion of the level of education attained by such personnel while ignoring promoting even the senior staff based on their long experience (number of years spent) at work and actual job performance even if such senior staff might had less qualifications. In contrast, the COs and nurses acknowledged the staff promotion criterion based on one's education or professional qualifications and job performance records rather than dependence on one'syears spent at work and favouritism of any other kind that are not guided by generally acceptable criteria as proposed by some of the workers. Thus, these controversial views were considered to have possible influence on the varied nature of the performance of frontline HWs in the provision of the desired services by quantity/volume and quality at hf level and that this applies to all services in general and even if examined for specific categories of the services such as IPTp or ANC. In other words, it was generally viewed that obviously the attitude HWs have had did reflect the trust/mistrust in the staff promotion and course attendance procedures for career development and this partly demotivated the HWs concerned to perform their duties as required by the national guidelines and as desired/expected by their clients. Giving an example, a mistrust was expressed regarding the in-charges of HFs who receive letters of invitation from the office of the DMO specifying the staff cadres to be nominated for attending on-the-job seminars or short courses related to their routine job of delivering RCH services, but such leaders coming to appoint the staff they like because of being driven by their own biases or negative attitudes to other eligible staff. As argued, this tendency is common in the private sector where the owners/in-charges of HFs do fear of the possibility of losing the staff who after acquiring additional skills/qualifications might be tempted to shift to other working places, including developing a desire for transferring to the public sector. Allegations against discriminatory and biased nomination of staff attending seminars were also described in terms of the indicated elements of nepotism or friendship between the nominated staff and their nominators (in-charges or seminar organizers) or agreement reached for sharing allowances acquired at seminars with some course organizers or leaders/superiors at work-places. This concern was much more raised by the respondents found in Mkuranga than in Mufindi, and it is interesting that the respondents insisted that this incidence should be brought openly to the attention of the authorities and other stakeholders concerned with training programmes for HWs in the country as a whole, as there is possibility that this has been experienced in other countries. Furthermore, in both districts concerns were raised about the tendency of some seminar/training organizers to select same individual staff each time the opportunity for training especially seminars and workshops comes. It was revealed further that in several cases, un-administrative personnel at district hospital level were surprised to note the district CHMT members and senior hospital nursing officers tending to nominate themselves for participating in seminars/workshops purposely as a way of using such an opportunity to maximize income in terms of earning the allowances usually offered to the training participants, thus leaving their junior (sub-ordinate) staff members who are the ones directly engaged in RCH services with fewer or no chances. A similar concern was also expressed among the lower level HF staff cadres including Medical Attendants and Health Assistants, as represented by the following quoted statement: "*We are the ones who deal with pregnant women more than MCHA who attend seminars, so if we are not selected for such seminars, what do they expect from us*?" (a Medical Attendant at Mwalusembe Evangelical Lutheran Church Dispensary, Mkuranga). This concern was shared even by the trained/skilled personnel including the Cos and nurses in both districts, thus the proposed solution to this problem being that there should be a centrally organized and funded upgrading of qualifications of Medical Attendants as is for the rest of the staff cadres.

Respondents from private health HFs considered colleagues from public facilities as being favoured for long-term career development: the following statement represents common views expressed by several other respondents: "*They give priority to staff working in government health facilities while we deliver services to the community and sometimes even more than they do in government facilities*" (HWs at private-for-profit clinics, Mkuranga). It was also revealed that employers in the private sector were reluctant to allow their staff to attend long-term training as a way of avoiding adhering to the government policy/law requiring the employers to pay higher salaries and related allowances after a worker has acquired additional qualification(s). CHMT members and HF in-charges in both districts supported the concerns expressed by the frontline HWs about private facility owners (especially those run by FBOs) paying their HWs less salaries than those paid to their public sector counterparts with the same academic/professional qualifications and experiences. This has been one of the factors reported to be demoralizing the private sector HWs among whom some have decided to shift to the public sector and therefore leaving a service delivery gap in the private sector.

In attempt to find out the truth about the concerns expressed by the private sector agencies regarding the training opportunities allocated for the private sector agencies versus public sector agencies, consultation was made through personal communications with the officers at the NMCP, particularly the Manager and Deputy Manager of the NMCP as well as the Head of Malaria In Pregnancy and Case Management of Malaria Unit. It was noted that the Tanzanian government has a policy that encourages/emphasizes (on) career development for all human resources who are employed in public and private sectors to be given opportunities and financial resources be available and the need for all the employers in both the public and private sectors to value this policy by taking the appropriate actions. That is why the staff working in the private sector have also been given a chance to participate in training programmes related to malaria control even if still there prevails a feeling of dissatisfaction of level of involvement of the private sector in public health training programmess as compared to the public sector, and this is not only in relation to malaria but also in relation to other public health programmes.

### Drug supply

Even when the HWs wished to deliver the IPTp services free-of-charge as the policy requires [19], it was occasionally not practically possible due to drug shortages. The shortage of SP required for IPTp frequently was reported by only 7(9.0%) of the respondents from both districts. Shortages were also reported to have been experienced in relation to antipyretics such as paracetamol, panadol and diclofenac and as reported these are usually administered to clients who report feeling uncomfortable after taking SP. As it was reported, these antipyretics were more permanently available for delivery free of user charges to the ANC clients in public HFs than in private facilities where the clients normally procure them as they occasionally do for SP given for IPTp unless there is any support from the government through the district health authorities. Respondents from the private sector were concerned about tendency of district authorities to ignore the private service providers when distributing the supplies of cold drugs (including SP for IPTp) as they normally do periodically to public HFs on ground that private providers would sell the drug to their clients for IPTp. This testimony about the latter to have been happening in the district(s) was given by the respondents as also confirmed by district CHMT members and national level officers. While such officers appreciated the need for the government to support private care providers with essential materials including (at least SP for IPTp) as the government recommends IPTp services to be delivered free of charge to the users and private-public-partnership in health service issues, the expressed doubt about the ability of private care providers to comply with the guideline requiring them to deliver IPTp free of charge [22].

### Health facility infrastructure, staff residential houses and other essential material conditions

Findings from descriptive analysis indicated that HW satisfaction with conditions of HF infrastructure and staff houses varied slightly between and even within the two districts, but the differences were not statistically significant for the cases reported herein on specific elements of infrastructure (data on p-values not shown). Because of the smallness nature of the data obtained for specific individual elements, the data from both districts were analyzed in combination. Conditions related to the physical infrastructure such as HF buildings and staff residential houses were assumed to affect staff motivation of the staff concerned even if indirectly when linked to IPTp services. Fourteen (18.2%) out of all respondents from both districts reported being satisfied with the conditions of their residential houses, the rest expressed some complaints mainly in relation to the size of the houses (rooms), general wearing and tearing of the buildings (poor condition of repair), or as reported by 19 (24.7%) of the respondents, lack of housing altogether despite government obligations to make staff housing available. Thirty-six (46.2%) out of 78 respondents were satisfied with the conditions of the consultation room at the HFs they were working while the rest reported shortage of office space and lack of privacy during clinical examinations. The rooms were considered small and noisy especially during MCH services when women were delivering babies and children crying. Speaking confidentially with clients was therefore perceived being difficult at such facilities. Among the 44 respondents among those who were working in HFs that were reported to be equipped with a laboratory, 18 (40.9%) were satisfied with the conditions of such facility while 26 (59.1%) found the space being too limited. Opinions on the availability of furniture at the ANC clinics where they were working were obtained from 77 interviewees whereby only 42 (55.3%) perceived the furniture to be insufficient (and majority of these respondents came from Mkuranga district). Forty-two (56.0%) out of 75 respondents were satisfied with the toilet facilities (for both clients and staff) whereas the remaining respondents had reservations related to inadequacy of water or general wear and tear. Mainly at peripheral dispensaries in Mkuranga, the presence of toilets without water, toilet paper, roofs and doors were reported and observed and these were reported to be part of the disappointments even to the MCH service clients. Staff and clients at such facilities in Mkuranga district therefore were forced to relieve themselves in the bush and this was a real concern as the residents in areas such as *Sotele, Kisiju-Kalole, Mkuranga town *and *Binga *where lions are commonly scavenging and occasionally killing people. Serious inadequacy of water at the HFs was reported by 50 (64.9%) out of 77 interviewees who gave response on this issue and these mainly came from Mkuranga district, whereas water was abundant according to the remaining respondents. Shortage of electricity supplies resulted in unreliable water supplies even at hospitals and health centres as the electrical pumps failed to push the water in the tanks and this increased the burden to the HWs who had to boil the water for use by the IPTp clients and sterilizing the syringes using firewood or charcoal in the absence of kerosene at HF level. Twenty-nine (37.2%) out of all 78 respondents perceived the ANC clinics available to be adequately equipped with cups for clients to take SP under DOT system. However, sharing the cups between successive IPTp dose users attended at the clinics was also reported to occasionally happen on days when there are many clients attending clinic and that this was viewed to be an inconvenience since it could be avoided if disposable cups were supplied to minimize the workload on the part of the HWs of fetching water for use by the clients to wash the cups after use so that the cups can be used by the next users. In contrast, the rest of the respondents found the cups being inadequate for IPTp users at their duty stations. Other materials and equipment such as weighing scales and blood pressure devices were reported to insufficiently available or were in poor condition of convenient use mainly at dispensary levels and this problem was reported in both districts. Respondents found working at the facilities owned by the FBOs were relatively more satisfied with conditions of buildings and equipment at their workplaces than their public facility respondents. Also, the respondents working at remote dispensaries seemed to be less satisfied with the conditions of the existing HF buildings than those found elsewhere in both districts (data not shown, but also researchers' direct observations of such conditions confirmed this to be the case). However, even the respondents found at the Mkuranga District Hospital owned by the government were not happy with the physical condition of their office buildings in terms of repair and office space at MCH clinic units. In Mufindi, a number of the dispensaries were undergoing renovation during the study implementation and this was reported to be a potential factor for improving the working morale of the HWs and attitudes of the potential ANC clients since a good health infrastructure is one of the attractions for service utilization. Working or living in poorly built or renovated and equipped buildings was perceived to negatively, even though indirectly, affect the staff's morale at their job places. In both districts, respondents were not fully satisfied with the so called 'government's endless promises to improve the physical working conditions including staff residential houses, office space, water and electricity supplies, medical equipment and other working conditions. The dissatisfaction with office space was explained in terms of lack of privacy during clinical consultations or physical examinations especially at dispensary levels, and this together with poor conditions of the toilets usually shared between staff and the clients are either poorly roofed or having no cemented floors or unsupplied with water for washing or having no toilet papers, precipitates even blames from the clients who consider that the HFmanagement or government authorities are irresponsive and negligent to the safety needs of patients.

Results from bivariate regression analyzes (Table 3) indicated somewhat controversial observations and this may be due to some of factors discussed critically later under the study limitations section in the 'Discussion' section presented later below.

It was noted that compared to the respondents found in private HFs, those found in public HFs were less likely to be satisfied with the working conditions, particularly in relation to: residential houses, HF buildings and furniture, availability of drinking and washing water, availability of electricity for boiling water for use by clients taking SP doses for IPTp under DOT and other purposes including lighting at evening/night times when there are emergency service conditions, shortage of medicines for IPTp, cups for use by clients taking IPTp doses under DOT, supply of lab materials and toilets for the staff and MCH service clients. Out of these observations, significant differences were only noted in relation to: satisfaction with availability and condition of residential houses (OR = 0.09; *p *= 0.001; CI: 0.02-0.39); furniture both at HF level in general (OR = 0.22; *p *= 0.004; CI: 0.08-0.62) and ANC clinic unit level within a particular HF (OR = 0.16; *p *= 0.001; CI: 0.06-0.45), laboratory building facilities (OR = 0.29; CI: *p *= 0.030; 0.09-0.89), water supply (OR = 0.32; 0.021; CI: 0.12-0.84), and availability of cups (OR = 0.09; *p *= 0.001; CI: 0.03-0.26), electricity supply at HF (0R = 0.32; *p *= 0.028; CI: 0.12-0.88).

## Discussion

### General overview of the key messages from the present study

Although it may sound difficult to separate the services related to IPTp administration as distinct from those of a general ANC service nature (given the fact that IPTp is one of the ANC services usually pregnant women seek at HFs, the present study reveals that HWs might have been appreciating IPTp as an essential intervention for malaria prevention for pregnant women attending ANC clinics and as part of safe motherhood strategy. However, such respondent showed a varied working morale that had consequent effects on the volume and quality of the services delivered-be it for RCH services in general or specifically for IPTp within the MCH clinic settings. The observed differences in HWs' motivation are partly contributed by the perceived variations in the working conditions such staff were facing at their work-places in terms of the physical conditions of the HF building state of repair, furniture, clean water and electricity supply, residential houses, availability of essential drugs and other supplies including working gears, as well as in relation to remunerations, office space and basic facilities such as furniture on one hand and other non-financial (e.g. moral) incentives including being recognized at work for one's performance, promotion, nomination to attend on-job training/seminars, and how friendly the supervision process is carried out by the superior officers at HF and district levels. In event of the constrained skilled and motivated manpower, it is not uncommon to find the existing HWs failing to comply with the service delivery guidelines. As discussed elsewhere by the present authors [22], the disappointment expressed by mostly respondents from the private sector on the issue of opportunities for training in relation to national focused ANC services as well as in relation to inadequate (almost non-existent) district support in terms of supply of essential medicines including IPTp for free service delivery to target clients marks another indicator of the possible failure of private sector providers to comply with the administration of IPTp doses as stipulated in the national(and WHO's) fANC guidelines.

HWs would not be happy working in situation whereby they experience blames from their clients in relation to quality of the services for reasons not caused by the service providers themselves: for instance, the reported shortages of medicines, water and laboratory diagnostic facilities, toilets and long waiting time at HF levels due to HW shortages, seem to be chronic challenges in the Tanzanian district health care systems in relation to ANC services including IPTp [19,22-24]. The reported sub-optimal effective implementation of fANC services in HFs caused by understaffing is likely to continue forcing the few available frontline HWs to deliver the services in haste with the view to completing their daily duties including filling in the health management information registers (MTUHA), cleaning HFs and yet having to attend emergency cases at evening times. By so doing, HWs may find themselves receiving blames from their clients who feel the services were given in rush with less courtesy or incompletely, leave alone those who may feel ubale to tolerate waiting longer for services when the existing HWs are busy because of being occupied with other routine services. Addressing the water shortage problem may not be a critical issue if concerted measures are taken to set budget for supplying bottled water to HFs for IPTp users [19] or establishing means that can help the staff at the facilities concerned harvest rain water and preserve it or the district government authorities liaising with potential donors to build a permanent water well for each HF.

### Strengths and limitations/weaknesses of the present study

Evidently, motivation is partly influenced by material incentives as well as moral incentives also matter [25,26], even if one cannot see the direct association of such material ownership/acquisition and the delivery of specific health services. Hanson *et al. *[11] suggest that assessment of HWs' morale/motivation using various (e.g. regression) models should be looked at from different angles of all possible contributing factors rather than looking at a single factor (e.g. s those used in the bivariate analysis in this paper), but again this depends on the way the model has been designed and the characteristics of the respondents as well as the HWs' (respondents') ability to understand the question and provide response. The present study has attempted to assess HWs' motivation for delivering IPTp services for malaria based on variables with quantitative dimensions such as HWs' satisfaction with conditions of remuneration, housing, HF infrastructure and essential supplies, and partly the variables with qualitative dimensions such as HWs feeling of being recognized for their job including being given opportunities for further career development and being supervised well for their routine service duties. This analysis, however, could not capture other potential factors for motivation some of which are psychological in nature in such a way that they could not be known in advance to be explained and included in the regression analyzes even though critics may be raised by other experts on this issue. It is evident that HWs' feeling lowly remunerated is likely to influence them work with low morale and this affects negatively the quality of the services delivered in general and for specific interventions such as IPTp. The latter point is valid in that the lessons can be learnt from the experience of doctors and paramedical personnel in a number of countries (including Tanzania) occasionally complaining to governments and reaching a point of boycotting to deliver services at least for some time (one can refer a recent boycott of doctors and supporting staff at the national and other hospitals in Tanzania as reported by different newspapers, radios and other mass media channels owned by the government and private organizations in 2011). The untimely or inadequate remuneration for HWs is often caused by the under-funding situation facing the government or health programme authorities, not necessarily negligence of the authorities concerned in developing countries [27]. The expressed concerns about limited opportunities for training as one of the key issues that have raised a sense of non-recognition among some of the HWs reflects the chances that those dissatisfied with the situation had felt less motivated to work harder under other constrained conditions, although this could not be explicitly explained by the logistic regression models used in the present study. Various authors emphasize that staff training is motivational factor for human resources for health to work harder and has an effect on the quality of the services delivered [28-30].

According to Manongi *et al. *[24], there have been limited studies and reports on HW motivational issues in relation to specific health interventions in Tanzania. The present study contributes by providing at least some evidence establishing the motivational factors that do actually (or have potential to) influence HWs' performance in terms of delivery of IPTp services against malaria given the contemporary environments in two rural districts. An alternative to constrained budgets that may not allow giving opportunities for training to all the available HWs in the health system would to strengthen HF supervision whereby the HWs would get an opportunity for being better oriented to particular service issues. Whether due to budget shortages or negligence by CHMT to carry out supportive supervision of HWs as reported in the present study, experts recommend regular supervision of frontline staff to give them feedback and a sense of feeling of to be recognized for their work and that this has a positive bearing including enhancing the HWs' working morale and performances [21,31,32].

As indicated by the quantitative results especially from regression analyses, there are mixed observations on the relevance of the inferences drawn based on the statistical tests employed regarding the issue of HW motivation and performance for a specific intervention like IPTp within a general MCH/ANC service delivery package and setting. Therefore, this analysis calls for further debate on, and where necessary, the most appropriate study design, implementation and analytical methodologies such as clear definitions of dependent and predictor variables for testing statistical associations in relation to determinants of IPTp service delivery. The analysts can consider a fair representation of private and public HFs as well as the sizes of the samples targeted to be involved from different cadres of health personnel and types of health facilities, geographical locations, etc. and this should be a basis for coming up more plausible research-based conclusions and policy recommendations in the future. This would allow focus to be made on other factor for inclusion in the analytical model designed with specific interventions implemented within a comprehensive service delivery package done for which the same cadres of the HWs are responsible to comply with.

The study findings based on the experience and views shared by the HWs who had been selected from only two districts and involving some convenient sampling technique. In addition, the majority of the regression analysis results presented are based on testing the association between a dependent variable and one predictor variable. Probably a more parsimonious regression model if used would have provided more realistic results that would minimize those that seemed counter-intuitive or controversial with those obtained from qualitative analysis. For instance, the opposite observation regarding the lowly skilled HWs expressing to be more satisfied with the working environment in the delivery of the general services and specifically IPTp than the skilled HWs could be explained by the fact that the more educated people are the more likely they are equipped with the capacity to observe/think about things critically based on their experience with previous or the current situation or both situations, leave alone the possible causes of regression model design and sampling design effects that might have affected the observed results. Also, often test of associations between variables using regression models sometimes ignore or underscore other potential variables that could explain the relationship much better, although even a so-considered most parsimonious model might still indicate some degree of imperfections since certain aspects are psychological in nature and cannot be quantified mathematically or statistically [20,33]. Care should also be taken to consider the results and inferences presented from the analyses made due to the possible design effects associated with the data presented in the present paper given the fact that different levels and numbers of HFs owned by different authorities (private -for-profit, FBO and public/government) as well as different categories and numbers of HWs were involved.

## Conclusions

Interestingly, HWs' indication of knowledge and appreciation of the IPTp strategy gives a good indication of the IPTp intervention strategy to be well recognized and this allows possible effective implementation should basic conditions be available in the district health care system. Therefore, to maximize its potential benefits, the worries expressed in terms of shortages related to HWs, essential supplies, buildings, administrative deficiencies and logistical difficulties in the health system should be addressed. The respondents' suggestions call for a close synergy (partnership) between the public and private sectors. This includes having proper arrangements for collaborative staff training programme on MCH/RCH services, shared supply of essential medicines and other materials to support the services, improvement of health infrastructure, strengthening supervision of frontline HWs (even under tight budgets since this may rise the need for more money to accomplish the plans e.g. for transport and HWs' compensation in terms of field allowances in peripheral HFs) and ensuring that HFs are adequately equipped with skilled/qualified health service personnel. The training opportunities could be ensured and supervised by higher levels of government (district or region) perhaps by keeping training rosters, and checking they are adhered to. The challenge is that despite the presence of the national policy emphasizing public-private-partnership (PPP) in various sectors including health, the real world practice of such a policy is inadequate and this is due to lack of a comprehensive policy and legal and institutional framework that provide clear guidelines and procedures for development and implementation of PPP [34]. Further studies involving a large number study participants (including HWs of different cadres by number and skills) selected with a much more randomized fashion from different geographical strata/clusters and involving the so considered more realistic approaches for distinguishing the qualitative issues from the quantitative issues or showing their linkages are recommended.

## Competing interests

The authors declare that they have no competing interests.

## Authors' contributions

All the listed co-authors commented substantially on the paper and were part of the design of the research proposal and research instruments. GMM was the principal investigator in the study that was part of his PhD training in health sciences with a specialty in the health economics, management, planning, and policy systems related to IPTp for malaria that was carried out under the Faculty of Health Sciences at the University of Copenhagen in Denmark. GMM has since 1996 been working the National Institute for Medical Research (NIMR) in Tanzania until now. All authors read and approved the final manuscript.
